# Reduced insulin action in muscle of high fat diet rats over the diurnal cycle is not associated with defective insulin signaling

**DOI:** 10.1016/j.molmet.2019.04.006

**Published:** 2019-04-12

**Authors:** Lewin Small, Amanda E. Brandon, Benjamin L. Parker, Vinita Deshpande, Azrah F. Samsudeen, Greg M. Kowalski, Jane Reznick, Donna L. Wilks, Elaine Preston, Clinton R. Bruce, David E. James, Nigel Turner, Gregory J. Cooney

**Affiliations:** 1 Diabetes and Metabolism Division, The Garvan Institute of Medical Research, Sydney, NSW, Australia; 2 The University of Sydney, School of Medical Science, Charles Perkins Centre D17, Sydney, NSW, Australia; 3 The University of Sydney, School of Life and Environmental Science, Charles Perkins Centre D17, Sydney, NSW, Australia; 4 Department of Pharmacology, School of Medical Science, University of New South Wales, Sydney, NSW, Australia; 5 Deakin University, School of Exercise and Nutrition Sciences, Faculty of Health, Institute for Physical Activity and Nutrition, Geelong, Australia

**Keywords:** Insulin action, Glucose uptake, Skeletal muscle, Insulin signaling, Diurnal rhythms, Phosphoproteomics

## Abstract

**Objective:**

Energy metabolism and insulin action follow a diurnal rhythm. It is therefore important that investigations into dysregulation of these pathways are relevant to the physiology of this diurnal rhythm.

**Methods:**

We examined glucose uptake, markers of insulin action, and the phosphorylation of insulin signaling intermediates in muscle of chow and high fat, high sucrose (HFHS) diet-fed rats over the normal diurnal cycle.

**Results:**

HFHS animals displayed hyperinsulinemia but had reduced systemic glucose disposal and lower muscle glucose uptake during the feeding period. Analysis of gene expression, enzyme activity, protein abundance and phosphorylation revealed a clear diurnal regulation of substrate oxidation pathways with no difference in Akt signaling in muscle. Transfection of a constitutively active Akt2 into the muscle of HFHS rats did not rescue diet-induced reductions in insulin-stimulated glucose uptake.

**Conclusions:**

These studies suggest that reduced glucose uptake in muscle during the diurnal cycle induced by short-term HFHS-feeding is not the result of reduced insulin signaling.

## Highlights

• Investigating metabolism in rodents over the diurnal cycle more accurately models normal animal physiology.
• Diurnal regulation of substrate oxidation is altered in muscle of HFHS-fed rats.
• There is a disconnect between glucose uptake and canonical insulin signaling in muscle.
• Activation of Akt2 does not rescue diet-induced reductions in insulin-stimulated glucose uptake in muscle.

## 1 Introduction

There is considerable research linking lipid-induced insulin resistance to defects in the canonical insulin signaling pathway. Both the diacylglycerol [1] and ceramide [2] lipid species have been hypothesized to interact with components of the insulin signaling pathway, commonly leading to a reported reduced phosphorylation and activity of Akt. This is then thought to result in the reduced phosphorylation of downstream targets of Akt including AS160 (involved in GLUT4 translocation to the plasma membrane) and GSK3β (an inhibitor of glycogen synthase) [3]. Due to the conflicting reports that observe either reduced [4], [5], [6] or no change [7], [8], [9], [10], [11] in Akt phosphorylation/activity in the muscle of insulin resistant or type 2 diabetic individuals under hyperinsulinemic-euglycemic clamp conditions, we concluded that it would be informative to interrogate insulin signaling and action over the normal feeding/fasting cycle.

Energy metabolism in mammals is regulated by mechanisms that follow a clear diurnal rhythm both at the whole-body level and in metabolically important tissues. For example, humans are active and eat mainly during the day, and rodents are most active during the night and eat the vast majority of their food in this phase. Assessing glucose metabolism and insulin action for any dysregulation relevant to the risk of developing type 2 diabetes at a single point in time therefore provides only a limited picture of the 24-hour physiology of glucose homeostasis. In rodent studies, interventions are often performed at a time that is convenient for the researcher, which is not necessarily the most physiologically relevant time to study glucose homeostasis in animals. Even controlling for plasma insulin concentrations, rodents have been shown to have substantial variation in insulin sensitivity over the diurnal cycle [12], [13].

In a different approach, we decided to investigate the relationship between circulating insulin, insulin signaling, and glucose metabolism *in vivo* over multiple points of the normal diurnal cycle without infusing exogenous glucose or insulin to control glycemia and insulinemia. This provided a situation where experimental animals only utilize substrates derived from digestion or energy stores at times that are relevant to their normal feeding/fasting behavior. By utilizing radioactive glucose tracers and collecting tissue samples over the diurnal cycle, we were able to measure glucose uptake rates, assess patterns of insulin signaling, gene transcription, and activity of metabolic enzymes in muscle under natural, physiological settings. The results show that reduced glucose uptake in the muscle of HFHS-fed rats over the diurnal cycle was not associated with reduced Akt signaling. We also provide evidence that increasing Akt activity by overexpression of a constitutively active Akt2 construct in muscle does not alter insulin-stimulated glucose uptake in the muscle of HFHS-fed rats

## 2 Material and methods

### 2.1 Animals

All surgical and experimental procedures performed were approved by the Garvan Institute/St. Vincent's Hospital Animal Ethics Committee and in accordance with the National Health and Medical Research Council of Australia's guidelines on animal experimentation. Male Wistar rats (150 g body weight at the start of feeding, Animal Resources Centre, Perth, Australia) were communally housed (3–4 to a cage) in temperature controlled (22 ± 0.5 °C), 12 h light–dark cycle rooms (light cycle, 7:00–19:00). Rats were fed *ad libitum* a standard chow diet (calorically, 14% fat, 67% carbohydrate, 19% protein, Rat Maintenance Diet; Specialty Feeds, Glen Forrest, Australia) or a lard-based high fat, high sucrose diet (HFHS; calorically, 46% fat, 34% carbohydrate {17% sucrose}, 20% protein) made in-house, for 4 weeks (exact dietary composition of the HFHS diet is detailed in Table S1, composition of the rat maintenance chow diet can be found from Specialty Feeds, Australia). This investigation utilized three separate cohorts of Wistar rats held under identical conditions. Rats in cohort 1 were utilized for the investigation of plasma metabolites and energy intake and expenditure every 3 h. Plasma samples were taken directly from the venous cannula from each animal immediately prior to sacrifice. Rats in cohort 2 were utilized for the investigation of glucose disposal, metabolic characterization in muscle at 4 timepoints (16:00, 21:00, 00:00 and 8:00) and proteomics analysis. Rats in cohort 3 were utilized for the *in vivo* electroporation and hyperinsulinemic-euglycemic clamp experiments. Rats were implanted with dual chronic cannulae in both jugular veins or in the right jugular vein and left carotid artery (hyperinsulinemic clamp experiments) [14], [15]. Animals were allowed one week to recover from surgery and were required to be within 5% of pre-surgical body weight to be included in experiments.

### 2.2 Respirometry, activity and energy intake

Whole body respirometry was performed on rats from cohort 1 after 3 weeks of diet (before surgery) utilizing the Columbus Oxymax indirect calorimetry system (Columbus Instruments, Columbus, OH, USA). Rats were individually housed and acclimatised for 2 days in Oxymax cages and food and water was available *ad libitum*. The airflow to each chamber was 1.2 L/min and O_2_ and CO_2_ measurements were taken every 15 min across a 48–72 h period and was averaged for every hour. Energy expenditure in kJ/hr/rat was calculated from VO_2_ and RQ measurements using the Lusk equation [16]. Activity was calculated by the sum of x, y, and z beam breaks, hourly in the Oxymax cages. Energy intake was measured by weighing of food hoppers and spillage in the home cages of the rats and correcting to account for multiple rats per cage and energy density of the diet (Chow 13 kJ/g, HFHS 20.4 kJ/g), for this measurement n number denotes separate cages.

### 2.3 In vivo glucose tracer disappearance

After recovering from surgery and regaining their pre-operative weight, chow and HFHS fed rats (cohort 2) were randomly assigned to groups for glucose tracer disappearance experiments. At one of the following times 16:00, 21:00, 00:00, 8:00 (corresponding to Zeitgeber time (ZT) ZT 9, ZT 14, ZT 17, ZT 1), rats were removed from the holding room and their cannulae were extended for unrestrained handling. Animals were conscious and able to move freely throughout the experiment, under dim light. A blood sample was taken via the left cannula and a bolus of trace amounts of 2-deoxy[^3^H]glucose and [^14^C]glucose was administered via the right cannula corresponding to ∼ 100 μCi/animal of each glucose tracer. Blood samples were taken over a period of 45 min to measure blood/plasma glucose and tracer concentrations. Larger samples of blood were taken at 0, 15, 30 and 45 min for determination of plasma insulin. 3.2% sodium citrate was used to clear the cannulae instead of heparin due to the effects of heparin on triglyceride lipolysis. After 45 min, the animals were infused with a lethal dose of pentobarbitone (60 mg/kg) to euthanize the animal. Tissues were rapidly dissected and freeze-clamped in liquid nitrogen prioritizing muscle.

### 2.4 In vivo electroporation

During the cannulation surgery in a third cohort of chow and HFHS-fed rats, the right tibialis cranialis (TC) muscle of each HFHS rat was electroporated with a pcDNA3 myr-HA-Akt2 plasmid while the contralateral muscle was electroporated with a control plasmid (pcDNA3-EH114-GFP or pcDNA3-luciferase). The pcDNA3 myr-HA-Akt2 construct codes for human Akt2 with an added N-terminal myristoylation sequence. This anchors Akt2 to the plasma membrane allowing for constitutive phosphorylation and activation by PDP1 and MTORC. The *in vivo* electrotransfer procedure has been previously described [17]. pcDNA3 myr-HA-Akt2 was a gift from William Sellers (Addgene plasmid # 9016). pcDNA3-EH114-GFP was subcloned previously [17]. pcDNA3-luciferase was a gift from William Kaelin [18] (Addgene plasmid # 18964). *In vivo* imaging of rats electroporated with pcDNA-luciferase was conducted one-week post electroporation using the IVIS Spectrum (Perkin Elmer, Waltham, MA) after intraperitoneal injection with 150 mg/kg D-luciferin.

### 2.5 Hyperinsulinemic-euglycemic clamp

For the caAKT2 experiment, one week post electroporation, animals were subjected to a 5 h fast commencing at 08:00 before starting a 2.5 h hyperinsulinemic-euglycemic clamp protocol in the conscious state (infusions started at 13:00) with a bolus of 2-deoxy[3H] glucose tracer administered once plasma glucose levels reached steady state as has been previously described [15]. The glucose infusion rate (GIR) was determined as the rate of glucose infusion once the animal had reached a steady state glucose concentration.

### 2.6 Biochemical analysis

Blood glucose during the clamp was determined via glucometer (Accu-Chek Perfoma; Roche Diagnostics, Castle Hill, Australia) and plasma glucose was determined by glucose oxidase assay (Infinity GOx; Thermo Fisher, Scoresby, Australia). Plasma insulin was measured by ELISA (mouse ultra-sensitive; Crystal Chem Inc, Elk Grove Village, USA) or radioimmunoassay (RIA; Linco Research Inc, St Charles, USA) and leptin, adiponectin, and corticosterone were determined by RIA (Linco Research Inc). Non-esterified fatty acid (NEFA) in the plasma was determined by NEFA C kit (Wako Diagnostics, Mountain View, USA). Tissue triglyceride was determined as glycerol equivalents after lipid extraction by the Folch method [19].

### 2.7 Tracer analytical methods

Plasma and tissue levels of ^3^H and ^14^C-labeled glucose tracers were measured to calculate whole body glucose disposal rate (R_d_), to estimate tissue glucose uptake (Rg'), and to measure incorporation of glucose into glycogen. Assays and calculations for glucose disappearance, glucose uptake into tissues and glucose incorporation into glycogen were performed as previously described [20].

### 2.8 Immunoblotting

Frozen, powdered tissue was homogenized by probe homogenizer in RIPA buffer containing protease inhibitor cocktail (1 × EDTA free; Roche) and phosphatase inhibitors (10 mM NaF, 1 mM Na_3_VO_4_). Tissue lysates were subjected to SDS-PAGE, transferred to PVDF membranes, blocked in 2% BSA and then immunoblotted with antibodies for pAkt Ser 473 (#9271), pan Akt (#9272), pAS160 Thr642 (#4288), AS160 (#2670) pGSK3β Ser9 (#9336) and GSK3β (#9315) (Cell Signaling Technology, Beverly, USA). Total amount of each protein was quantified on the same membranes as phospho-proteins after stripping (0.5 M NaOH, 30 min) and reprobing. Densitometry analysis was performed using ImageJ software (NIH; http://imagej.nih.gov/ij/).

### 2.9 Enzyme activity assays

Hexokinase, citrate synthase, beta-hydroxyacyl-CoA dehydrogenase (β-HAD), and pyruvate dehydrogenase (PDH) assays were conducted spectrophotometrically. Frozen, powdered muscle was homogenized in 20 volumes of 50 mM Tris-HCl, 1 mM EDTA, 0.1% Triton X-100, pH 7.4 by probe homogenizer on ice. Homogenates were freeze/thawed in three cycles between liquid nitrogen and a 30 °C water bath. Homogenates were centrifuged at 4700 g for 10 min at 4 °C and the supernatant was transferred to a new tube. All assays were conducted in a plate reader (SpectraMax Plus 384) at 30 °C in a 300 μl final volume. Muscle homogenate was incubated with the reaction buffer before assay initiation at 30 °C for 3 min to deplete endogenous substrate. The following conditions were used for each assay.

Hexokinase: The reaction mixture was 50 mM imidazole, 9 mM ATP, 9 mM MgCl_2_, 0.6 mM NADP^+^, excess glucose-6-phosphate dehydrogenase, pH 7.4. 20 μl of 1/20 homogenate, and 230 μl of reaction buffer was added to each well. The reaction was initiated with 50 μl of 30 mM glucose in excess reaction buffer. The reaction was followed at 340 nm for 5 min.

Citrate Synthase: The reaction mixture was 100 mM Tris-HCl, 1 mM MgCl_2_, 1 mM EDTA, 1 mM DTNB (Ellman's Reagent), 0.4 mM acetyl-CoA, pH 8.2. 10 μl of 1/120 homogenate and 240 μl of reaction buffer was added to each well. The reaction was initiated with 50 μl of 3 mM oxaloacetate in excess reaction buffer. The reaction was followed at 412 nm for 5 min.

β-HAD: The reaction mixture was 50 mM imidazole, 1.2 mM EDTA, 0.18 mM NADH, pH 7.4. 10 μl of 1/20 homogenate, and 240 μl of reaction buffer was added to each well. The reaction was initiated with 50 μl of 0.6 mM acetoacetyl-CoA in excess reaction buffer. The reaction was followed at 340 nm for 5 min.

PDH: PDH activity was determined in both the endogenous and fully activated state. 10 mM NaF and 1 mM sodium dichloroacetate were included in the homogenization buffer for this assay. The reaction mixture was 100 mM Tris-HCl, 1 mM MgCl_2_, 1 mM EDTA, 0.2 mM CoA, 1 mM NAD^+^, 1 mM thiamine pyrophosphate, 50 μM 4-Aminoazobenzene-4′-sulfonic acid, excess arylamine acetyltransferase (extracted from pigeon liver acetone powder, Sigma, as previously described [21]), pH 7.8. 30 μl of 1/20 homogenate and 220 μl of reaction buffer was added to each well. The reaction was initiated with 50 μl of 10 mM sodium pyruvate in excess reaction buffer. The reaction was followed at 390 nm for 30 min. Full activation of PDH was achieved by a 30-minute incubation of muscle homogenate with 1.5 mM CaCl_2_ and 20 ng/μl of recombinant human pyruvate dehydrogenase phosphatase 1 (PDP1, Abcam) at 37 °C in the absence of NaF.

Glycogen synthase activity was assayed radiometrically as previously described [22].

### 2.10 RNA extraction, cDNA synthesis and RT-PCR

RNA was extracted from frozen, powdered muscle using the phenol/chloroform method, with Tri reagent (Sigma). cDNA was synthesized from 2 μg of DNAse-treated RNA using Qiagen's Omniscript RT kit and random 9mers (NEB). Muscle gene expression was analyzed with Universal Probe Library (UPL, Roche) probes, oligos designed by UPL Assay Design Center and LightCycler ® 480 Probes Master mastermix (Roche) using the LightCycler 480 system and software utilizing primers listed in Table S2. The following PCR protocol was used: The initial activation step (95 °C for 10 min) was followed by 40 repetitions of the cycling stage (95 °C 20 s denaturation, 60 °C 40 s annealing, 72 °C 20 s extension) and a final cooling step (40 °C 30 s). Relative quantification was determined by the addition of a standard curve of pooled cDNA for each gene in each run. Technical triplicates were run for each sample for each gene and averaged. Relative gene expression was normalized to the housekeeping gene, *ppia* (cyclophilin A).

### 2.11 Polar metabolite extraction and GC-MS analysis

Frozen muscle was weighed (∼10–20 mg) in 2 ml safety lock Eppendorf tubes and kept on dry ice prior to extraction. Extraction solution consisting of a Chloroform: Methanol: Water mix in a 1:3:1 (v/v/v) ratio, respectively was mixed with Scyllo-inositol internal standard stock (10 mM; 2 μl per 750 μl extraction solution). Briefly, 750 μl of ice cold extraction/internal standard solution was added to each sample tube, including extraction blanks (i.e. tubes with no tissue), with all samples being kept on ice throughout extraction procedure. Samples were homogenized using a mechanical hand homogenizer. Samples were vigorously vortexed and allowed to incubate on ice for 10 min followed by 10 min centrifugation at maximum speed (0 °C). The supernatant was transferred to fresh Eppendorf tubes on ice and 300 μl milli-Q water added to each tube (ratio of chloroform: methanol: water now 1:3:3) after which the samples were again vortexed vigorously and centrifuged at 0 °C for 5 min (5000×*g*). Following centrifugation, 200 μl of upper aqueous phase was collected and transferred into a GC vial and insert and then dried in a speed vacuum at 30 °C. The dried extracts were then methoximated by the addition of 20 μl methoxyamine (Sigma; 20 mg/ml in pyridine) and vials capped, vortexed and derivatized at 37 °C for 2 h. Following methoximation, samples underwent TMS derivatization by the addition of 20 μl BSTFA + 1% TCMS (Thermo Fisher), vortexed and incubated at 60 °C for 30 min. Metabolites were analyzed on an Agilent 6890N GC system and an Agilent 5975C MSD (Agilent Technologies, Santa Clara, CA, USA) in the electron ionization (EI) mode, with helium as the carrier gas. A VF-5 capillary column with 10 m inert EZ-guard (J&W Scientific, 30 m, 0.25 mm inner diameter, 0.25 μm film thickness) was used and the front inlet and transfer line temperatures were both set to 270 °C, while the quadrupole and source temperatures were set to 150 °C and 230 °C, respectively. Sample (1 μL) were injected in the splitless mode using the following oven gradient: 70 °C for 2 min; 12.5 °C/min to 295 °C followed by 25 °C/min to 320 °C with a final hold time of 3 min. The mass selective detector was operated in the scan mode (35–650 m/z). Specific details of the elution time for each metabolite as well as the fragmentation pattern and ions quantified can be provided on request and can also be found on the NIST database. The abundance of each chromatographic peak was calculated by integrating the area under the curve (AUC) for each metabolite specific ion using Agilent Mass Hunter Quantitative analysis software. Data are presented as relative intensities, i.e. metabolite ion AUC normalized divided by the internal standard ion AUC and finally divided by the tissue weight.

### 2.12 Phosphopeptide enrichment and MS

Approximately 30 mg of powdered red quadriceps tissue was homogenized in 400 μl of homogenization buffer (6 M urea, 2 M thiourea, 20 mM triethylammonium bicarbonate (TEAB)) with protease (1 × EDTA-free protease inhibitor cocktail, Roche) and phosphatase inhibitors (10 mM NaF, 1 mM Na_3_VO_4_) and 0.1% SDS. Protein digestion, tandem mass tag (TMT) labeling and phosphopeptide enrichment were performed as previously described [23]. Fractionation of mono-phosphorylated peptides was performed using hydrophilic interaction liquid chromatography (HILIC) over a 1 h gradient. Each TMT reaction was separated into 36 fractions that were concatenated in 12 fractions. Multi-phosphorylated peptides were run unfractionated due to the lower complexity (lower abundance). Total protein samples were fractionated using a HILIC column over the same 1-hour gradient and concatenated into 12 fractions. Phosphopeptides were analyzed on a Dionex 3500RS nanoUHPLC coupled to an Orbitrap Plus mass spectrometer in positive mode. Peptides were separated using an in-house packed 75 μm × 40 cm pulled column (1.9 μm particle size, C18AQ; Dr Maisch, Germany) with a gradient of 2–30% acetonitrile containing 0.1% FA over 120 min at 250 nl/min at 55 °C. An MS1 scan was acquired from 350 to 1550 (70,000 resolution, 3e6 AGC, 100 ms injection time) followed by MS/MS data-dependent acquisition with HCD (35,000 resolution, 2e5 AGC, 120 ms injection time), 32 NCE, 1.2 *m*/*z* quadrupole isolation width. Non-phosphorylated peptides for total proteome analysis were analyzed on a Dionex 3500RS nanoUHPLC coupled to an Orbitrap Fusion mass spectrometer in positive mode. Peptides were separated using identical chromatography conditions as above. An MS1 scan was acquired from 350 to 1550 (60,000 resolution, 4e5 AGC, 50 ms injection time) followed by MS/MS data-dependent acquisition with CID with detection in the ion trap (rapid scan rate, 2e4 AGC, 70 ms injection time, 35% NCE, 1.6 *m*/*z* quadrupole isolation width). A synchronous precursor selection and MS3 analysis was performed with HCD and detection in the orbitrap for quantification of reporter ions (selection of fragment ions 400–1000 *m*/*z*, 1e5 AGC, 105 ms injection time, 55% normalized collision energy, 2 *m*/*z* ion trap isolation width, orbitrap scanning width 100–500 *m*/*z*, [24]).

Raw MS files were processed with MaxQuant software (Version 1.5.8.3, Max Planck Institute of Biochemistry [25]) using the default settings including TMT labeling and phospho STY sites. Database searching was performed using MaxQuant's Andromeda search engine against the UniProt *Rattus norvegicus* reference proteome (Proteome ID UP000002494_10116). Peak intensities were normalized to a standard sample that was added to each run (pooled red quadriceps from 3 fasted chow-fed rats) and normalized difference was log 2 transformed. In order to normalize distribution between samples, expression values were subtracted by the median of the column they were in (log 2 expression values). Removal of unwanted variation method (RUV) was applied to remove any batch effects and identify differentially regulated phosphosites and proteins [26].

### 2.13 Statistical analysis

Diurnal rhythmicity in Figure 1 was determined using the MetaCycle R package and corrected for multiple comparison testing using the Fisher method [27]. Diurnal data with 4 timepoints (Figure 1, Figure 2) were analyzed by 2-way ANOVA for a main effect of time and a main effect of diet. Sidak's post-hoc test was used to determine statistical difference between diets at specific time-points. Results between chow and HFHS animals combining time-points (Table 1) were compared using an unpaired t-test. Analysis of phosphoproteomic and total proteomic data was primarily carried out in Perseus (Version 1.6, Max Planck Institute of Biochemistry [28]). Multiple comparison testing was carried out using the Benjamini-Hochberg method with a false discovery rate of 5%. In the Akt2 gain of function investigation, paired t-tests were used to compare contralateral limbs. All error bars are depicted as means ± SEM. n number for all experiments indicates samples from individual animals. Statistical analysis was performed in GraphPad Prism software (Prism 7). P < 0.05 was considered statistically significant.

### 2.14 Data availability

The proteomic and phosphoproteomic datasets have been deposited to the ProteomeXchange Consortium (http://proteomecentral.proteomexchange.org) via the PRIDE partner repository [29] with the dataset identifier <PXD010242>. Summarized phosphoproteomic and proteomic datasets with fold change and adjusted p values are available in the supplementary information (Table S4) as excel spreadsheets.

## 3 Results and discussion

### 3.1 Distinct effects of diet on the diurnal abundance of plasma parameters

After 4 weeks of HFHS-feeding, rats had significantly higher body and fat pad weights as well as higher muscle and liver triglyceride content compared to chow controls (Table 1). In order to select time-points for further investigation, chow and HFHS rats were observed for changes in plasma parameters and energy intake every 3 h over the diurnal cycle. The vast majority of food in both chow and HFHS fed rats was eaten during the dark (night) period with a small percentage (∼20%) being eaten during the day (light period) (Figure 1A). Similarly, energy expenditure increased during the night when the animals were active; however, there was no difference in energy expenditure between chow and HFHS rats (Figure 1B). The respiratory exchange ratio (RER) of chow fed rats displayed the expected diurnal rhythm in which carbohydrate was primarily oxidized during the night when animals were feeding, while fat oxidation increased during the day (Figure 1C). HFHS animals showed a significantly lower RER over the entire 24-hour period that lacked diurnal variation, indicating higher fat oxidation at all times. As expected, activity was increased during the dark cycle and was similar between chow and HFHS rats (Figure 1D). Plasma glucose levels were not significantly altered by time or diet (Figure 1E). Plasma NEFA displayed diurnal rhythmicity in both chow and HFHS rats but was higher in HFHS animals, particularly during the feeding period (Figure 1F). Circulating insulin was significantly rhythmic in both chow and HFHS animals and was highest during the dark phase (Figure 1G). Plasma leptin and adiponectin were higher in HFHS animals but only displayed significant diurnal variation in chow animals (Figure 1H,I). As expected, plasma corticosterone displayed a clear diurnal variation with levels rising during the day, peaking at 18:00 and then falling through the night (Figure 1J). There was no effect of diet on plasma corticosterone levels.

**Table 1:** Fat mass and tissue triglyceride content.

Diet	Chow	HFHS	Effect of Diet
Final Body Weight (g)	370 ± 4	389 ± 5	**
**Fat Pad Weights at Cull (g)**			
EpiWAT	4.2 ± 0.2	7.1 ± 0.4	****
RpWAT	4.5 ± 0.3	7.5 ± 0.4	****
IngWAT	5.1 ± 0.3	7.9 ± 0.4	****
**Tissue Triglyceride Content (μmol/g)**			
Tibialis Cranialis	5.1 ± 0.3	6.6 ± 0.4	***
Red Quadriceps	6.9 ± 0.3	8.9 ± 0.5	***
Liver	9.4 ± 0.7	21.9 ± 1.4	****

Values are means ± SEM. Analyzed by individual student's t-tests. n = 38. **p < 0.005, ***p < 0.005, ****p < 0.0001. ns = not significant. EpiWAT = epididymal white adipose tissue, Rp = retroperitoneal, Ing = inguinal.

**Figure 1:**
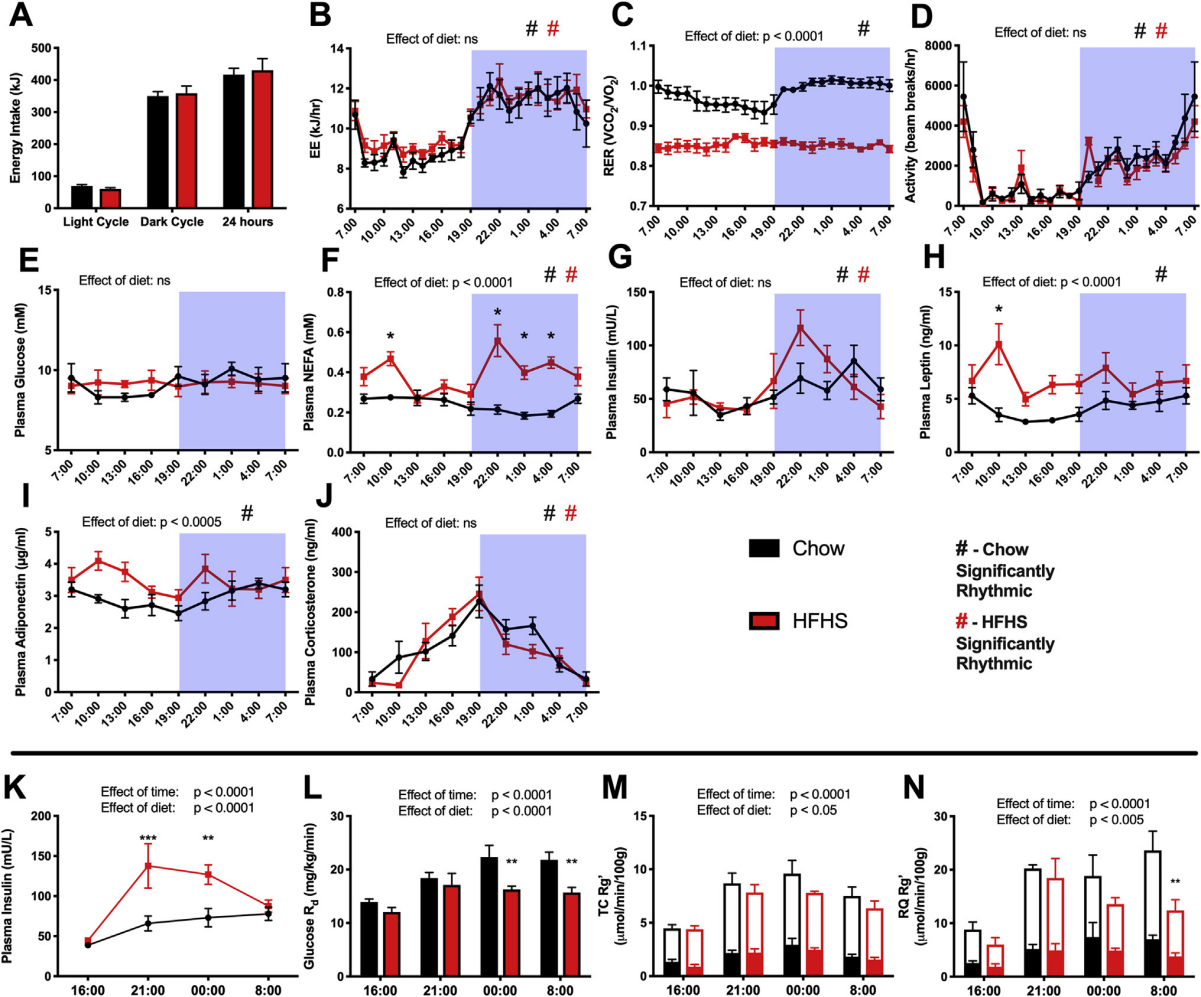
**Energy intake, expenditure, and plasma parameters display diurnal variation and glucose disposal and muscle glucose uptake is reduced in HFHS rats.** Black = Chow. Red = HFHS. (A) Energy intake, n = 4–6. (B) Energy expenditure. (C) Respiratory exchange ratio. (D) Activity. n = 5. 3 hourly measurements of (E) plasma glucose, (F) NEFA, (G) insulin, (H) leptin, (I) adiponectin, and (J) corticosterone. The 7:00 time-point is double-plotted in graphs D-J; however, this is not included in the statistical analysis. n = 4–12. Analyzed by Metacycle to test for rhythmicity and 2-way ANOVA for a main effect of diet. In a second cohort of animals, (K) plasma insulin. (L) Rate of glucose disappearance from plasma. Rate of glucose uptake (total bars) and proportion of this rate incorporated into glycogen (filled bars) in the (M) tibialis cranialis and (N) red quadriceps muscles. n = 5–10. Analyzed by 2-way ANOVA for a main effect of time and a main effect of diet. *p < 0.05, **p < 0.005, ***p < 0.0005. #p < 0.05 for rhythmicity. Data presented are mean ± SEM.

### 3.2 HFHS rats display reduced systemic and muscle insulin action over the diurnal cycle

Four time-points over the diurnal cycle were chosen to investigate glucose disposal in further detail. Rats were studied at 16:00, a time-point chosen due to the relatively little energy intake in rodents during the day (Figure 1A). 21:00 and 00:00 were chosen as time-points when the animals were actively feeding during the dark cycle and 8:00, one hour after lights switch back on. Correspondingly, the stomach weights from animals studied at 21:00, 00:00, and 8:00 were significantly heavier than those at 16:00 (Table S3). Due to the higher energy density of the HFHS diet, the similar stomach weights between chow and HFHS rats at 21:00 and 00:00 suggest that HFHS rats either had an increased energy intake or a delayed rate of gastric emptying during these times. Plasma glucose and insulin levels over the 45-minute radioactive glucose tracer disappearance experiment stayed relatively constant within groups (Fig. S1) and were therefore averaged for ease of interpretation.

Interestingly, animals had higher insulin levels at the feeding timepoints (Figure 1G,K; 19:00, 00:00), particularly HFHS-fed rats; however, animals did not display significantly higher glycemia (Table S3). This may indicate insulin release due to incretin changes in response to feeding rather than insulin release only reflecting changes in blood glucose levels. It is important to consider that the hyperinsulinemia in HFHS animals during the feeding period may be partly due to the sucrose content in the HFHS diet driving hyperinsulinemia compared to the more complex carbohydrate present in chow. A similar result was found in humans in which increasing doses of glucose during an oral glucose tolerance test induced larger insulin responses despite the same glycemic response [30].

Using this model, we were able to discern reduced insulin action both systemically and in muscle at multiple time-points over the diurnal cycle in HFHS rats. Despite exhibiting hyperinsulinemia (Figure 1K), HFHS rats displayed a lower rate of glucose disappearance from the circulation (R_d_; Figure 1L) and reduced glucose uptake into the muscle (Rg'; Figure 1M,N) compared to chow controls over the normal feeding period. Another parameter influenced by insulin, glucose incorporation into glycogen, increased in the feeding phases in both muscles but was significantly lower in the RQ of HFHS rats (Table S3). This demonstrated that HFHS rats were insulin resistant, both in the muscle and at the whole-body level, over the diurnal cycle. Glucose uptake, glucose incorporation into glycogen and glycogen content in other tissues are displayed in Table S3.

Correlating with the higher rate of glucose uptake, red quadriceps muscle from chow-fed animals tended to have a higher abundance of the glycolytic intermediate glucose-6-phosphate (G6P; p = 0.08) and had a significantly higher abundance of fructose-6-phosphate (F6P), which was most pronounced during the feeding time-points (Table 2). Muscle levels of alanine, serine, aspartate, and the branched chain amino acids valine and isoleucine were higher in muscle from HFHS rats. AMP and glutamate levels were highest at 16:00 and fell substantially during the feeding time-points; however, the amount of these metabolites was not affected by diet. Conversely, muscle lactate levels were the lowest at 16:00 and rose during the feeding time-points. Levels of the TCA cycle intermediates citrate, succinate, fumarate, and malate were not altered with diet or time.

**Table 2:** Relative concentrations of metabolites from red quadriceps muscle.

Time	16:00		21:00		00:00		Statistical tests	
Diet	Chow	HFHS	Chow	HFHS	Chow	HFHS	Effect of Time	Effect of Diet
G6P	1 ± 0.33	2.32 ± 2.01	4.63 ± 1.86	2.63 ± 1.35	9.46 ± 2.22	3.47 ± 1.55	*	p = 0.08
F6P	1 ± 0.15	1.03 ± 0.27	1.6 ± 0.11	0.84 ± 0.26	1.76 ± 0.35	1 ± 0.11	ns	*
Lactate	1 ± 0.13	1.07 ± 0.08	1.42 ± 0.08	1.04 ± 0.11	1.39 ± 0.11	1.49 ± 0.1	**	ns
AMP	1 ± 0.24	0.95 ± 0.1	0.61 ± 0.04	0.45 ± 0.07	0.35 ± 0.02	0.3 ± 0.03	****	ns
Alanine	1 ± 0.06	1.54 ± 0.19	1.16 ± 0.09	1.24 ± 0.12	1.02 ± 0.05	1.58 ± 0.19	ns	**
Glutamate	1 ± 0.04	1.06 ± 0.13	0.74 ± 0.06	0.77 ± 0.04	0.87 ± 0.05	0.82 ± 0.05	**	ns
Serine	1 ± 0.06	1.36 ± 0.1	0.98 ± 0.05	1.23 ± 0.1	1.03 ± 0.09	1.45 ± 0.23	ns	**
Glycine	1 ± 0.13	1.47 ± 0.16	1.75 ± 0.12	1.18 ± 0.21	1.2 ± 0.16	1.29 ± 0.28	ns	ns
Aspartate	1 ± 0.1	1.17 ± 0.19	0.61 ± 0.08	0.77 ± 0.09	0.57 ± 0.04	0.93 ± 0.06	**	**
Valine	1 ± 0.05	1.47 ± 0.06	1.32 ± 0.08	1.45 ± 0.18	1.34 ± 0.07	1.83 ± 0.16	*	***
Isoleucine	1 ± 0.09	1.51 ± 0.09	1.25 ± 0.11	1.43 ± 0.2	1.17 ± 0.06	1.69 ± 0.13	ns	**
Citrate	1 ± 0.12	1.53 ± 0.09	1.3 ± 0.07	1.18 ± 0.11	1.28 ± 0.07	1.5 ± 0.11	ns	ns
Succinate	1 ± 0.22	1.47 ± 0.21	1.38 ± 0.09	0.99 ± 0.16	1.06 ± 0.06	1.38 ± 0.14	ns	ns
Fumarate	1 ± 0.19	1.03 ± 0.16	1.4 ± 0.18	0.85 ± 0.15	1.36 ± 0.07	1.78 ± 0.48	ns	ns
Malate	1 ± 0.2	1.09 ± 0.13	1.19 ± 0.18	0.7 ± 0.12	1.17 ± 0.08	1.39 ± 0.28	ns	ns

Values are means ± SEM relative to chow 16:00. Analyzed by individual 2-way ANOVAs for an effect of time and an effect of diet. n = 4–6. *p < 0.05, **p < 0.005, ***p < 0.0005, ****p < 0.0001. ns = not significant.

### 3.3 Distinct effects of time and diet on the expression and activity of metabolic proteins

**Figure 2:**
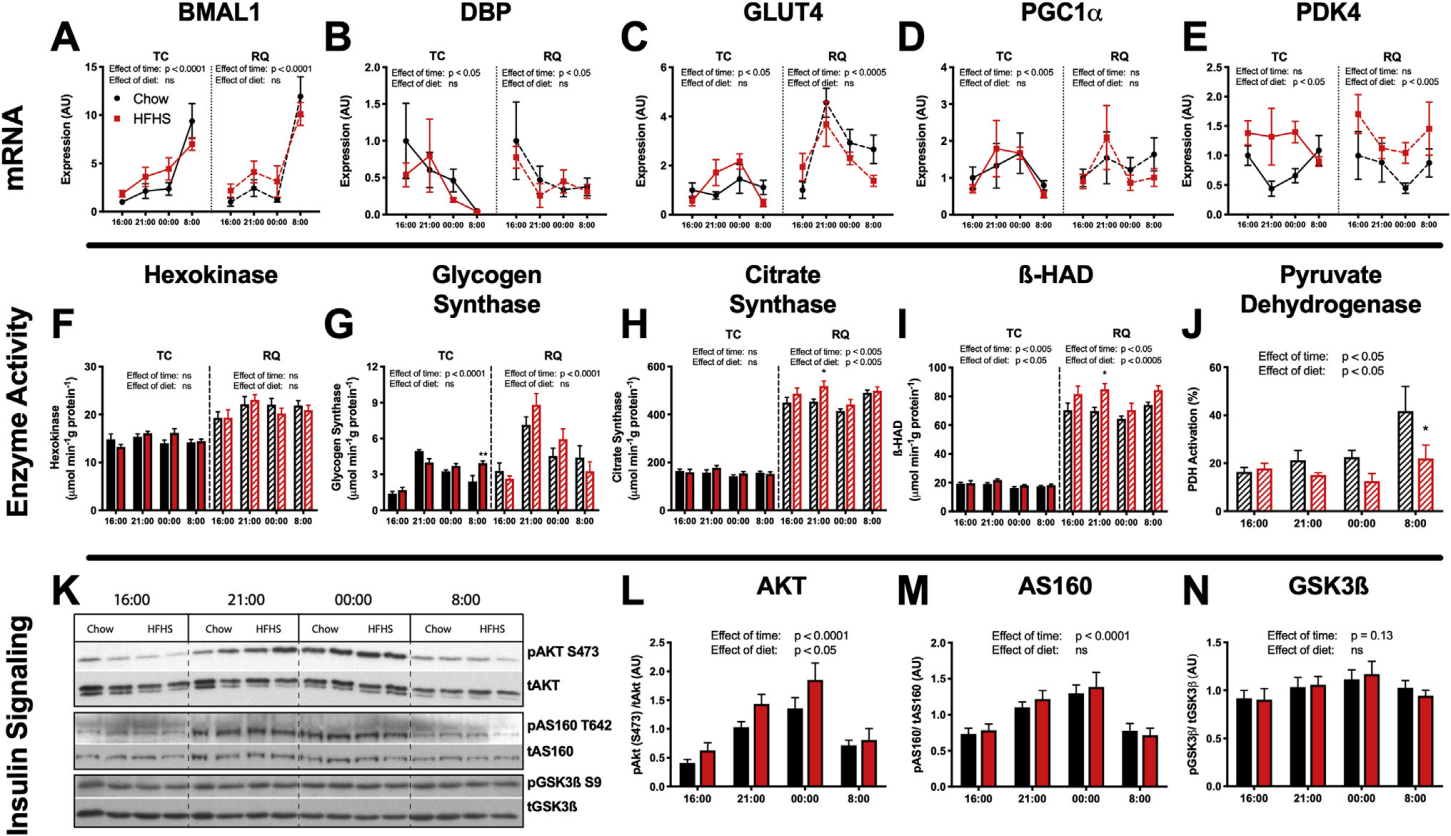
**Diurnal variation of mRNA abundance, enzyme activity and insulin signaling highlights increased fatty-acid oxidation in HFHS muscle compared to chow with no differences in Akt signaling.** Black = Chow. Red = HFHS. Solid lines/bars = TC. Dashed lines/hatched bars = RQ. Muscle mRNA expression of (A) BMAL1, (B) DBP, (C) GLUT4, (D) PGC1α and (E) PDK4, compared to the average expression in the chow 16:00 group for each muscle. n = 4–6. Muscle enzyme activities of (F) hexokinase, (G) glycogen synthase, (H) citrate synthase, (I) β-HAD and (J) pyruvate dehydrogenase. Active and maximal PDH activity were measured for the PDH assay and therefore it is displayed as % activation. n = 5–7. (K) Representative immunoblot and densitometry of (L) Akt (Ser 473), (M) AS160 (Thr 642) and (N) GSK3β (Ser 9) phosphorylation in TC muscle. n = 7–12. Analyzed by 2-way ANOVA for a main effect of time and a main effect of diet. *p < 0.05, **p < 0.005. Data presented are mean ± SEM.

There have been reports that high fat diet-feeding alters the rhythmic expression of core circadian genes (such as *dbp* and *bmal*) in the hypothalamus, liver, and adipose tissue and therefore could disturb normal diurnal feeding behavior and perhaps alter substrate metabolism [31]. In muscle, our data show a clear difference in the expression of the core circadian genes *bmal* and *dbp* across different times; however, no difference in expression between chow and HFHS diets (Figure 2A,B). These observations are consistent with previous literature [32] and support our conclusions that the current study is reporting metabolic events that align with the normal diurnal rhythms of fasting/feeding and substrate metabolism in rats. Disrupting clock machinery in muscle through genetic ablation of bmal1 has been reported to reduce both GLUT4 and hexokinase 2 gene expression [33]. In the current study, expression of GLUT4 mRNA in muscle showed circadian periodicity (although not to the extent of the core circadian regulatory genes); however, no effect of HFHS-feeding was observed (Figure 2C)

Glucose phosphorylation by hexokinase has been implicated as a potential rate limiting step responsible for impaired glucose uptake into muscle [34]. No difference was seen in hexokinase activity between diets or times in TC or RQ muscles in the current studies (Figure 2F), and although the level of G6P in muscle tended to increase over the feeding period it was not significantly different in HFHS muscle (Table 2). Together, this suggests that it is unlikely that hexokinase abundance or allosteric inhibition of hexokinase by G6P is responsible for the differences observed in insulin-stimulated glucose uptake. Glycogen synthase activity has a strong diurnal variation that tracks with glucose incorporation into glycogen and glucose uptake. Glycogen synthase activity was not significantly different between diets (Figure 2G); however, this *in vitro* measurement of enzyme activity may not accurately reflect the flux through this enzyme *in vivo*.

**Figure 3:**
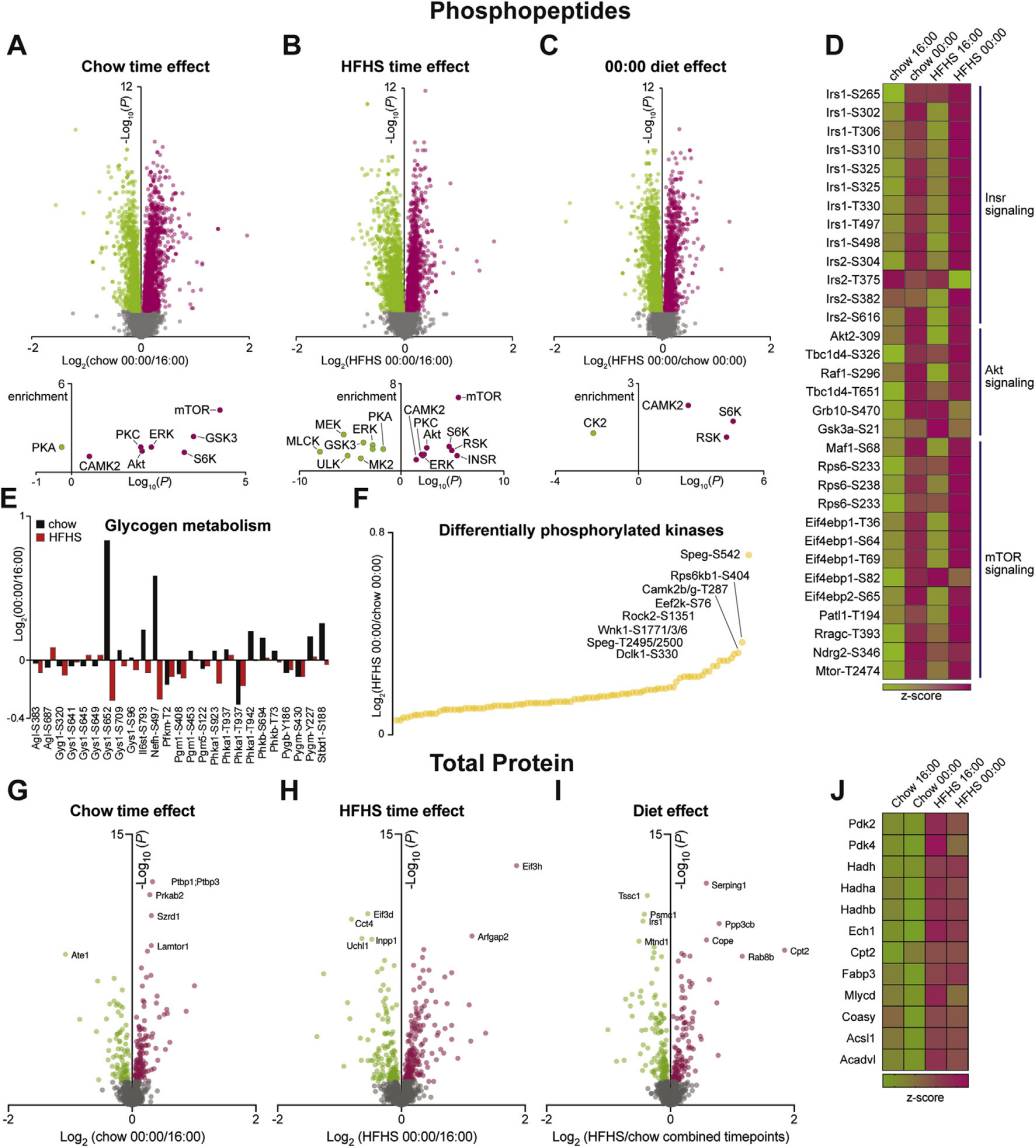
**Phosphoproteomic analysis of red quadriceps muscle reveals substantial diurnal variation and little effect of diet on the abundance of canonical insulin signaling phosphopeptides**. Volcano plots of phosphopeptide abundance and kinase enrichment analysis (A) comparing chow muscle between 00:00 and 16:00, (B) comparing HFHS muscle between 00:00 and 16:00 (C) and comparing chow and HFHS muscle from 00:00. (D) Heatmap of phosphosites involved in insulin receptor, Akt and mTOR signaling. (E) Abundance of phosphopeptides involved in glycogen metabolism comparing muscle between 00:00 and 16:00. (F) Kinases that show the greatest difference in phosphorylation between chow and HFHS muscle from 00:00. Volcano plots of total protein abundance (G) comparing chow muscle between 00:00 and 16:00, (H) comparing HFHS muscle between 00:00 and 16:00 (I) and comparing chow and HFHS muscle from both timepoints combined. (J) Heatmap of proteins involved in lipid metabolism that were significantly increased in HFHS muscle. Green = low abundance, red = high abundance. n = 3–7.

The lack of change in muscle PGC1α gene expression (Figure 2D) or citrate synthase activity (Figure 2H) in response to HFHS feeding across all time-points suggested that differences in mitochondrial number/density were not contributing to the development of insulin resistance in HFHS rats. However, ß-hydroxyacyl-CoA dehydrogenase (β-HAD), one of the enzymes in the β-oxidation pathway, did show a small but significant increase in activity in HFHS muscle (Figure 2I), consistent with previous reports [35], as well as an increase in the abundance of the subunits of β-HAD, HADH, HADHA, and HADHB (Figure 3J). These data indicate that the HFHS diet specifically up-regulates enzymes involved in fatty acid oxidation in muscle. The reduction in PDH activity in HFHS muscle (Figure 2J), presumably driven by the increase in pyruvate dehydrogenase kinase (PDK) 4 gene and protein abundance (Figure 2, Figure 3J), indicates that glucose oxidation was down-regulated by the high dietary fat content of the HFHS diet. A similar repression of muscle PDH activity has been reported in humans after both 3 days [36] and 6 weeks [37] of exposure to a high-fat diet. However, investigations by our group and others suggest that changes in PDH activity alone cannot explain the reductions in insulin-stimulated glucose uptake seen in muscle from HFHS fed animals [38], [39]. In summary, although mitochondrial density is not different between diets, there is a clear oxidative shift from glucose to fatty acid oxidation in the muscle of HFHS rats that could contribute to differences in insulin-stimulated glucose uptake and reflects the increased availability of fatty acids in the plasma after feeding (Figure 1F).

### 3.4 Akt signaling is similar in the muscle of chow and HFHS rats

The phosphorylation state of Akt, a key kinase in the insulin signaling pathway, as well as its downstream targets AS160 and GSK3β increased significantly across the feeding phase and was highest at the feeding time-points, 21:00 and 00:00 (Figure 2L). Importantly, there was no effect of diet on phosphorylation of Akt in muscle at any of the corresponding time-points at which glucose uptake was decreased (Figure 2L). Similarly, no differences in phosphorylation were seen in the downstream targets of Akt, AS160 (Figure 2M), and GSK3β (Figure 2N) in muscle across both diets despite the differences observed in glucose uptake. Although there were no differences found in phosphorylation between the muscle of chow and high HFHS rats, there was substantial hyperinsulinemia in HFHS animals at 21:00 and 00:00 (Figure 1K). Therefore, the argument can be made that the similar phosphorylation status of signaling proteins in the presence of higher insulin levels does reflect a decreased ability of insulin to stimulate insulin signaling pathways (insulin resistance). However, reduced glucose uptake at 21:00 and 00:00 despite no difference in the phosphorylation levels of signaling intermediates suggests that, irrespective of differences in insulin levels, Akt signaling cannot completely explain the differences in glucose uptake observed in muscle of HFHS-fed rats. Additionally, while there were elevated insulin levels at 21:00 and 00:00 in HFHS animals, insulin levels were relatively matched at 8:00 where there continued to be a lower glucose uptake in muscle from HFHS rats without any detectable difference in phosphorylation of Akt, AS160, or GSK3β. Our studies add to a growing number of reports that did not find differences in Akt signaling in the muscle of high fat diet fed rodents [38], [40], [41], [42] and suggest there are likely other mechanisms that regulate glucose uptake into muscle that are altered by a HFHS diet.

### 3.5 Substantial effect of time on the phosphorylation of insulin signaling intermediates with little effect of diet

The current study also provides a unique perspective on changes in phosphopeptides in muscle *in vivo* that occur when animals go through the normal fasting/feeding transition. Phosphoproteomic analysis was performed on RQ muscle from chow and HFHS animals at 16:00 and 00:00 with a total of 14,447 unique phosphorylation sites quantified (n = 3–7). As expected, substantial differences in the abundance of phosphopeptides were observed between 16:00 and 00:00 when food was consumed and circulating insulin levels increased in the muscle of both chow (1909 up, 1487 down; Figure 3A) and HFHS rats (1771 up, 1795 down; Figure 3B). Kinase enrichment analysis identified phosphopeptides in the mTOR, S6K, and Akt pathways as being significantly increased in muscle from rats at 00:00 (Figure 3A,B). When phosphopeptide abundance was compared between muscle from HFHS and chow-fed rats at 00:00, there were substantial significant differences (1369 up, 1490 down; Figure 3C), but interestingly there was no enrichment for insulin signaling or Akt pathways. A closer look at specific phosphopeptides involved in insulin receptor, Akt and mTOR signaling, showed a pattern of substantial differences with time but little effect of diet (Figure 3D). Despite few changes in the phosphorylation of canonical insulin signaling kinases, several phosphosites on proteins involved in glycogen metabolism had altered abundance between chow and HFHS muscle, including glycogen synthase 1 (GYS1; Figure 3E). Other interesting sites that were some of the most significant differentially regulated phosphosites between chow and HFHS muscle at 00:00 were from striated muscle preferentially expressed protein kinase (SPEG), S6K, and CAMK2B (Figure 3F). Interestingly, phosphorylation of CAMK2B at Thr287 was increased in muscle from HFHS rats at 00:00 compared to chow controls (Table S4). A similar finding has been reported in the muscle of insulin resistant humans [43].

Because the same individual muscle sample was used for both glucose uptake experiments and phosphoproteomic analysis, this data set was additionally analyzed to determine if abundance of phosphopeptides correlated significantly with plasma insulin levels and muscle glucose uptake independent of diet or time (Table S4). 96 phosphopeptides correlated with plasma insulin including well established, insulin-regulated phosphopeptides, IRS1 Ser307, PRAS40 Thr247, and p70S6K Ser427 [44] while 61 phosphopeptides correlated with glucose uptake including glycogen synthase Ser649. Total proteomic analysis comparing muscle from chow and HFHS rats identified 259 differentially abundant proteins, 124 that were significantly more abundant in HFHS muscle (Figure 3I). Many of these were involved in fatty acid metabolism (Figure 3J), including subunits of the β-HAD enzyme complex and PDK4. A full list of identified phosphopeptide and total protein abundance has been uploaded as a supplemental excel spreadsheet (Table S4). The lack of difference in the phosphorylation state of key insulin signaling proteins despite reduced glucose uptake in HFHS muscle suggests that factors other than phosphorylation are involved in regulating the activity of signaling proteins or that mechanisms other than alterations in canonical insulin signaling contribute to reduced muscle glucose uptake in response to excess dietary fat.

### 3.6 caAkt2 overexpression does not rescue lipid-induced defects in glucose metabolism

**Figure 4:**
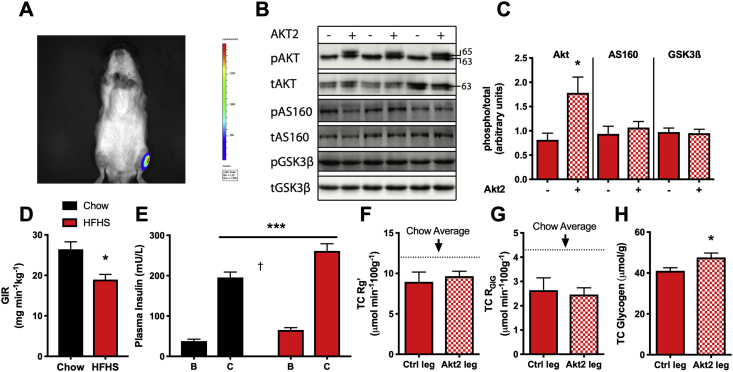
**Overexpression of a constitutively active Akt2 construct does not rescue lipid-induced insulin resistance in muscle**. Black = Chow. Red = HFHS. Solid = control leg. Checked = caAkt2 overexpressing leg (A) *In vivo* imaging system picture of luciferase transfection of the tibialis cranialis. (B) Representative immunoblot of the TC muscle from 3 rats ± caAkt2 plasmid. (C) Densitometry of Akt (Ser473), AS160 (Thr 642) and GSK3β (Ser 9) phosphorylation in HFHS TC muscle ± caAkt2 plasmid. (D) Glucose infusion rate during the hyperinsulinemic-euglycemic clamp, analyzed by t-test (E) [B] basal and [C] clamped plasma insulin, analyzed by 2-way ANOVA. (F) Glucose uptake between legs, pairwise comparison. (G) Glucose incorporation into glycogen between legs, pairwise comparison. Dotted line = average of chow muscle. n = 6. (H) Glycogen content between legs, pairwise comparison. n = 9. Comparisons between legs of individual rats were analyzed by paired t-test. *p < 0.05, ***p < 0.0005. †p < 0.0001 main effect of clamp. Data presented are mean ± SEM.

In order to further explore the role of Akt activity in lipid-induced insulin resistance in muscle we utilized a gain of function model to overexpress a constitutively active Akt2 (caAkt2) protein in muscle *in vivo*. Overexpressing of caAkt2 in muscle of chow fed rats has previously been shown to increase basal glucose uptake [17]. In the current study, Akt was overexpressed to determine if increasing Akt activity would rescue defects in insulin-stimulated glucose uptake in muscle caused by HFHS feeding. Figure 4A shows luminescence specifically in the left TC of a rat transfected with a pcDNA3-luciferase construct, without spill-over into other muscles after subcutaneous delivery of the luciferin substrate. Overexpression of caAkt2 was confirmed by immunoblotting for Akt at the serine 473 residue (Figure 4B,C). Phosphorylation of the caAkt2 construct distinct from endogenous Akt can be observed because of the slight size shift due to the added myristoylation tag on caAkt2 (63 kDa–65 kDa). On average, transfection resulted in ∼2-fold increase in total phosphorylation of Akt at serine 473 in the overexpressing leg compared to the control leg (Figure 4C). Interestingly, no differences were seen in the phosphorylation of AS160 and GSK3β between the caAkt2 expressing leg and control leg (Figure 4B,C). This lack of effect of increased Akt phosphorylation on phosphorylation of downstream targets is comparable with what was observed in muscle over the diurnal cycle where changes in phosphorylation of AS160 and GSK3β were small despite a relatively large dynamic range of Akt phosphorylation. A similar lack of correlation between changes in Akt phosphorylation and phosphorylation of its downstream targets has been observed in insulin-stimulated 3T3-L1 cells [45] and human muscle after a hyperinsulinemic-clamp [5]. When HFHS rats with caAkt2 and control transfected TC muscles and chow controls were subjected to insulin stimulation during a hyperinsulinemic-euglycemic clamp, HFHS rats had a significantly reduced glucose infusion rate (GIR) (Figure 4D) even though the clamp plasma insulin levels were slightly higher in HFHS animals (Figure 4E). Together this suggests that HFHS rats had systemic insulin resistance driven by a reduction in insulin-stimulated glucose disposal. In HFHS rats, both glucose uptake and glucose incorporation into glycogen under hyperinsulinemic-euglycemic clamp conditions were reduced compared to chow controls and no difference was observed between control and caAkt2 overexpressing muscles (Figure 4F,G) for either parameters. Interestingly, glycogen content was significantly higher in the caAkt2 leg of HFHS-fed animals, which may indicate a greater rate of glycogen synthesis under basal conditions (Figure 4H). This suggests that the caAkt2 construct has a functional role in glucose metabolism that is more evident in a non-insulin stimulated state which is consistent with a previous investigation [17]. Overall, these data indicate that overexpression of Akt2 to increase Akt activity did not rescue lipid-induced insulin resistance in muscle. This provides further evidence for the idea that changes in Akt activity are not the only factor responsible for lipid-induced defects in insulin-stimulated glucose uptake in muscle and simply measuring the phosphorylation state of Akt is not an appropriate surrogate for insulin-stimulated glucose uptake under all conditions.

## 4 Conclusions

In conclusion, the data presented in this paper show that over the normal diurnal cycle of fasting and feeding in rats there is a clear diurnal fluctuation in glucose uptake and phosphorylation of insulin signaling proteins in muscle. However, the reduced glucose uptake in muscle over this cycle, caused by relatively short-term feeding of a HFHS diet is not accompanied by reduced levels of phosphorylation of canonical insulin signaling proteins. Moreover, increasing the amount of activated Akt2 does not necessarily dictate insulin-stimulated glucose uptake into muscle. Clearly, canonical insulin signaling is required for normal insulin-stimulated glucose disposal in muscle as demonstrated by the large amount of literature based on the genetic disruption of insulin signaling components [46]. Similarly, it is possible that in the case of chronic obesity, in tandem with aging [47], or in individuals with particular genetic variants [48], insulin signaling can be compromised and make a major contribution to reduced insulin-stimulated glucose uptake. However, in the current study the pathways most altered by the HFHS diet were involved more directly with the metabolism of glucose and fatty acids rather than differences in phosphorylation of signaling proteins. The regulation of glucose uptake and metabolism in muscle is multifactorial and the circulating level of glucose [49], fatty acids [50], and hormones such as insulin all play a part. Similarly, reductions in insulin-stimulated glucose uptake may be an additive effect of defects in extracellular glucose and insulin delivery (blood flow, microvascular recruitment), glucose transport (glucose transporter number and activity), and phosphorylation (hexokinase activity) [34]. Therefore, when investigating mechanisms of lipid-induced insulin resistance, focusing on factors altering the canonical insulin signaling pathway may be limiting, particularly in the context of differences that occur over the normal diurnal feeding cycle rather than artificially controlled experimental conditions.

## Author contributions

Conceptualisation, L.S. and G.J.C., Methodology, L.S., A.E.B., B.L.P., V.D., G.M.K., J.R., D.L.W., E.P., N.T., and G.J.C. Investigation, L.S., A.E.B., B.L.P., A.S., G.M.K., J.R., D.L.W., E.P., and G.J.C. Writing original draft, L.S. and G.J.C. Writing, review and editing, L.S., A.E.B., B.L.P., G.M.K., C.R.B., D.E.J., N.T., and G.J.C. Funding acquisition, G.J.C. Resources, C.R.B., D.E.J., and G.J.C. Supervision, A.E.B., N.T., and G.J.C.

## Figures and Tables

**figs1:**
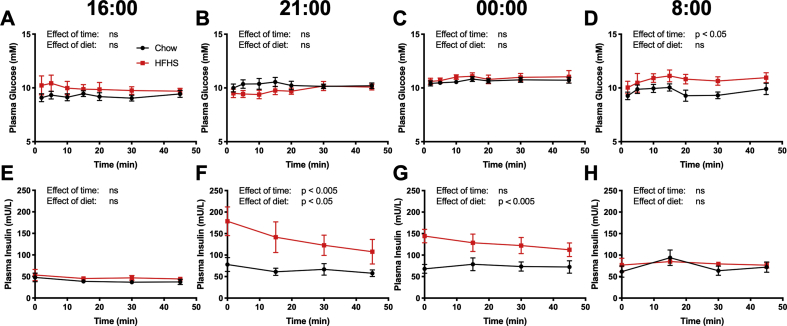


## References

[1] Szendroedi J., Yoshimura T., Phielix E., Koliaki C., Marcucci M., Zhang D. (2014). Role of diacylglycerol activation of PKCθ in lipid-induced muscle insulin resistance in humans. Proceedings of the National Academy of Sciences.

[2] Chavez J.A., Summers S.A. (2012). A ceramide-centric view of insulin resistance. Cell Metabolism.

[3] Froejdoe S., Vidal H., Pirola L. (2009). Alterations of insulin signaling in type 2 diabetes: a review of the current evidence from humans. Biochimica Et Biophysica Acta.

[4] Krook A., Roth R.A., Jiang X.J., Zierath J.R., Wallberg-Henriksson H. (1998). Insulin-stimulated Akt kinase activity is reduced in skeletal muscle from NIDDM subjects. Diabetes.

[5] Tonks K.T., Ng Y., Miller S., Coster A.C.F., Samocha-Bonet D., Iseli T.J. (2013). Impaired Akt phosphorylation in insulin-resistant human muscle is accompanied by selective and heterogeneous downstream defects. Diabetologia.

[6] Albers P.H., Pedersen A.J.T., Birk J.B., Kristensen D.E., Vind B.F., Baba O. (2015). Human muscle fiber type-specific insulin signaling: impact of obesity and type 2 diabetes. Diabetes.

[7] Kim Y.B., Nikoulina S.E., Ciaraldi T.P., Henry R.R., Kahn B.B. (1999). Normal insulin-dependent activation of Akt/protein kinase B, with diminished activation of phosphoinositide 3-kinase, in muscle in type 2 diabetes. Journal of Clinical Investigation.

[8] Højlund K., Staehr P., Hansen B.F., Green K.A., Hardie D.G., Richter E.A. (2003). Increased phosphorylation of skeletal muscle glycogen synthase at NH2-terminal sites during physiological hyperinsulinemia in type 2 diabetes. Diabetes.

[9] Bandyopadhyay G.K., Yu J.G., Ofrecio J., Olefsky J.M. (2005). Increased p85/55/50 expression and decreased phosphotidylinositol 3-kinase activity in insulin-resistant human skeletal muscle. Diabetes.

[10] Beeson M., Sajan M.P., Dizon M., Grebenev D., Gomez-Daspet J., Miura A. (2003). Activation of protein kinase C-zeta by insulin and phosphatidylinositol-3,4,5-(PO4)3 is defective in muscle in type 2 diabetes and impaired glucose tolerance: amelioration by rosiglitazone and exercise. Diabetes.

[11] Meyer M.M., Levin K., Grimmsmann T., Beck-Nielsen H., Klein H.H. (2002). Insulin signalling in skeletal muscle of subjects with or without Type II-diabetes and first degree relatives of patients with the disease. Diabetologia.

[12] Basse A.L., Dalbram E., Larsson L., Gerhart-Hines Z., Zierath J.R., Treebak J.T. (2018). Skeletal muscle insulin sensitivity show circadian rhythmicity which is independent of exercise training status. Frontiers in Physiology.

[13] Shi S.-Q., Ansari T.S., McGuinness O.P., Wasserman D.H., Johnson C.H. (2013). Circadian disruption leads to insulin resistance and obesity. Current Biology CB.

[14] Hoy A.J., Brandon A.E., Turner N., Watt M.J., Bruce C.R., Cooney G.J. (2009). Lipid and insulin infusion-induced skeletal muscle insulin resistance is likely due to metabolic feedback and not changes in IRS-1, Akt, or AS160 phosphorylation. American Journal of Physiology Endocrinology and Metabolism.

[15] Bakshi I., Suryana E., Small L., Quek L.-E., Brandon A.E., Turner N. (2018). Fructose bisphosphatase 2 overexpression increases glucose uptake in skeletal muscle. Journal of Endocrinology.

[16] Kaiyala K.J., Morton G.J., Leroux B.G., Ogimoto K., Wisse B., Schwartz M.W. (2010). Identification of body fat mass as a major determinant of metabolic rate in mice. Diabetes.

[17] Cleasby M.E., Reinten T.A., Cooney G.J., James D.E., Kraegen E.W. (2007). Functional studies of Akt isoform specificity in skeletal muscle in vivo; maintained insulin sensitivity despite reduced insulin receptor substrate-1 expression. Molecular Endocrinology (Baltimore, Md.).

[18] Safran M., Kim W.Y., O'Connell F., Flippin L., Günzler V., Horner J.W. (2006). Mouse model for noninvasive imaging of HIF prolyl hydroxylase activity: assessment of an oral agent that stimulates erythropoietin production. Proceedings of the National Academy of Sciences of the United States of America.

[19] Folch J., Lees M., Sloane Stanley G.H. (1957). A simple method for the isolation and purification of total lipides from animal tissues. Journal of Biological Chemistry.

[20] Kraegen E.W., James D.E., Jenkins A.B., Chisholm D.J. (1985). Dose-response curves for in vivo insulin sensitivity in individual tissues in rats. American Journal of Physiology.

[21] Tabor H., Mehler A.H., Stadtman E.R. (1953). The enzymatic acetylation of amines. Journal of Biological Chemistry.

[22] Oakes N.D., Cooney G.J., Camilleri S., Chisholm D.J., Kraegen E.W. (1997). Mechanisms of liver and muscle insulin resistance induced by chronic high-fat feeding. Diabetes.

[23] Hoffman N.J., Parker B.L., Chaudhuri R., Fisher-Wellman K.H., Kleinert M., Humphrey S.J. (2015). Global phosphoproteomic analysis of human skeletal muscle reveals a network of exercise-regulated kinases and AMPK substrates. Cell Metabolism.

[24] McAlister G.C., Nusinow D.P., Jedrychowski M.P., Wühr M., Huttlin E.L., Erickson B.K. (2014). MultiNotch MS3 enables accurate, sensitive, and multiplexed detection of differential expression across cancer cell line proteomes. Analytical Chemistry.

[25] Cox J., Mann M. (2008). MaxQuant enables high peptide identification rates, individualized p.p.b.-range mass accuracies and proteome-wide protein quantification. Nature Biotechnology.

[26] Gagnon-Bartsch J.A., Jacob L., Speed T.P. (2013). Removing unwanted variation from high dimensional data with negative controls.

[27] Wu G., Anafi R.C., Hughes M.E., Kornacker K., Hogenesch J.B. (2016). MetaCycle: an integrated R package to evaluate periodicity in large scale data. Bioinformatics (Oxford, England).

[28] Tyanova S., Temu T., Sinitcyn P., Carlson A., Hein M.Y., Geiger T. (2016). The Perseus computational platform for comprehensive analysis of (prote)omics data. Nature Methods.

[29] Vizcaíno J.A., Côté R.G., Csordas A., Dianes J.A., Fabregat A., Foster J.M. (2013). The PRoteomics IDEntifications (PRIDE) database and associated tools: status in 2013. Nucleic Acids Research.

[30] Kowalski G.M., Moore S.M., Hamley S., Selathurai A., Bruce C.R. (2017). The effect of ingested glucose dose on the suppression of endogenous glucose production in humans. Diabetes.

[31] Kohsaka A., Laposky A.D., Ramsey K.M., Estrada C., Joshu C., Kobayashi Y. (2007). High-fat diet disrupts behavioral and molecular circadian rhythms in mice. Cell Metabolism.

[32] Reznick J., Preston E., Wilks D.L., Beale S.M., Turner N., Cooney G.J. (2013). Altered feeding differentially regulates circadian rhythms and energy metabolism in liver and muscle of rats. Biochimica Et Biophysica Acta.

[33] Dyar K.A., Ciciliot S., Wright L.E., Biensø R.S., Tagliazucchi G.M., Patel V.R. (2014). Muscle insulin sensitivity and glucose metabolism are controlled by the intrinsic muscle clock. Molecular Metabolism.

[34] Wasserman D.H. (2009). Four grams of glucose. American Journal of Physiology Endocrinology and Metabolism.

[35] Turner N., Bruce C.R., Beale S.M., Hoehn K.L., So T., Rolph M.S. (2007). Excess lipid availability increases mitochondrial fatty acid oxidative capacity in muscle: evidence against a role for reduced fatty acid oxidation in lipid-induced insulin resistance in rodents. Diabetes.

[36] Lundsgaard A.-M., Sjøberg K.A., Høeg L.D., Jeppesen J., Jordy A.B., Serup A.K. (2017). Opposite regulation of insulin sensitivity by dietary lipid versus carbohydrate excess. Diabetes.

[37] Lundsgaard A.-M., Holm J.B., Sjøberg K.A., Bojsen-Møller K.N., Myrmel L.S., Fjære E. (2019). Mechanisms preserving insulin action during high dietary fat intake. Cell Metabolism.

[38] Small L., Brandon A.E., Quek L.-E., Krycer J.R., James D.E., Turner N. (2018). Acute activation of pyruvate dehydrogenase increases glucose oxidation in muscle without changing glucose uptake. American Journal of Physiology Endocrinology and Metabolism.

[39] Rahimi Y., Camporez J.-P.G., Petersen M.C., Pesta D., Perry R.J., Jurczak M.J. (2014). Genetic activation of pyruvate dehydrogenase alters oxidative substrate selection to induce skeletal muscle insulin resistance. Proceedings of the National Academy of Sciences.

[40] Brandt N., De Bock K., Richter E.A., Hespel P. (2010). Cafeteria diet-induced insulin resistance is not associated with decreased insulin signaling or AMPK activity and is alleviated by physical training in rats. American Journal of Physiology Endocrinology and Metabolism.

[41] Turner N., Kowalski G.M., Leslie S.J., Risis S., Yang C., Lee-Young R.S. (2013). Distinct patterns of tissue-specific lipid accumulation during the induction of insulin resistance in mice by high-fat feeding. Diabetologia.

[42] De Vogel-van den Bosch J., Hoeks J., Timmers S., Houten S.M., van Dijk P.J., Boon W. (2011). The effects of long- or medium-chain fat diets on glucose tolerance and myocellular content of lipid intermediates in rats. Obesity (Silver Spring, Md.).

[43] Højlund K., Birk J.B., Klein D.K., Levin K., Rose A.J., Hansen B.F. (2009). Dysregulation of glycogen synthase COOH- and NH2-terminal phosphorylation by insulin in obesity and type 2 diabetes mellitus. Journal of Clinical Endocrinology & Metabolism.

[44] Humphrey S.J., Yang G., Yang P., Fazakerley D.J., Stöckli J., Yang J.Y. (2013). Dynamic adipocyte phosphoproteome reveals that Akt directly regulates mTORC2. Cell Metabolism.

[45] Hoehn K.L., Hohnen-Behrens C., Cederberg A., Wu L.E., Turner N., Yuasa T. (2008). IRS1-independent defects define major nodes of insulin resistance. Cell Metabolism.

[46] Nandi A., Kitamura Y., Kahn C.R., Accili D. (2004). Mouse models of insulin resistance. Physiological Reviews.

[47] Sharma N., Arias E.B., Sajan M.P., MacKrell J.G., Bhat A.D., Farese R.V. (2010). Insulin resistance for glucose uptake and Akt2 phosphorylation in the soleus, but not epitrochlearis, muscles of old vs. adult rats. Journal of Applied Physiology (Bethesda, Md.: 1985).

[48] Latva-Rasku A., Honka M.-J., Stancáková A., Koistinen H.A., Kuusisto J., Guan L. (2017). A partial loss-of-function variant in AKT2 is associated with reduced insulin-mediated glucose uptake in multiple insulin sensitive tissues: a genotype-based callback positron emission tomography study. Diabetes.

[49] Alzaid A.A., Dinneen S.F., Turk D.J., Caumo A., Cobelli C., Rizza R.A. (1994). Assessment of insulin action and glucose effectiveness in diabetic and nondiabetic humans. Journal of Clinical Investigation.

[50] Høeg L.D., Sjøberg K.A., Jeppesen J., Jensen T.E., Frøsig C., Birk J.B. (2011). Lipid-induced insulin resistance affects women less than men and is not accompanied by inflammation or impaired proximal insulin signaling. Diabetes.

