# Supplementary material for: Reduced insulin action in muscle of high fat diet rats over the diurnal cycle is not associated with defective insulin signaling
Source: Mol Metab. 2019 Apr 12;25:107–18. doi: 10.1016/j.molmet.2019.04.006 (PMC6600078; doi:10.1016/j.molmet.2019.04.006)
Supplement: Multimedia component 1 [file mmc1.docx]

**Supplementary information titles and legends**

Figure S1: **Plasma glucose and insulin over the tracer disappearance period from rats at 16:00, 21:00, 00:00 and 8:00.** (A-D) Plasma glucose. (E-H) Plasma insulin. Analyzed by repeated measures 2-way ANOVA. n = 5-10. Data presented are mean ± SEM.

Table S1: **High fat high sucrose diet formula**

|  | Grams per kg | kJ per kg | Energy (% total) |
| --- | --- | --- | --- |
| casein | 230 | 3845 | 18.8 |
| sucrose | 202 | 3388 | 16.6 |
| starch | 170 | 2843 | 13.9 |
| mineral mix (AIN76) | 45 |  |  |
| trace minerals (AIN93) | 13 |  |  |
| bran | 50 | 587 | 2.9 |
| methionine | 3 |  |  |
| gelatine | 20 | 339 | 1.7 |
| choline | 4 |  |  |
| safflower oil | 30 | 1127 | 5.5 |
| lard | 220 | 8287 | 40.6 |
| vitamin mix (AIN76) | 13 |  |  |
| total | 1001 | 20416 | 100 |
| protein | 250 | 4184 | **20** |
| carbohydrate | 423 | 6818 | **34** |
| fat | 250 | 9414 | **46** |

| Table S2: **Real time PCR primers** | | | |
| --- | --- | --- | --- |
| **Gene Name** | **Direction** | **Primer Sequence** | **UPL Probe #** |
| *arntl* (BMAL1) | Forward | tccgatgacgaactgaaaca | 74 |
|  | Reverse | cggtcacatcctacgacaaa |  |
| *dbp* | Forward | ttctgcagggaaacagcaa | 94 |
|  | Reverse | ccttgcgctccttttcct |  |
| *ppargc1a* (PGC1α) | Forward | aaagggccaagcagagaga | 29 |
|  | Reverse | gtaaatcacacggcgctctt |  |
| *slc2a4* (GLUT4) | Forward | ttgcagtgcctgagtcttctt | 120 |
|  | Reverse | ccagtcactcgctgctga |  |
| *pdk4* | Forward | gagctgttctcccgctacag | 120 |
|  | Reverse | agttctctcacaggcattttctg |  |
| *ppia* (Cyclophillin A) | Forward | ttgctgcagacatggtcaa | 156 |
|  | Reverse | tgtctgcaaacagctcgaag |  |

| Table S3: **Glucose disposal in rats over the diurnal cycle** | | | | | | | | | | |
| --- | --- | --- | --- | --- | --- | --- | --- | --- | --- | --- |
| **Time** | **16:00** | | **21:00** | | **00:00** | | **8:00** | | **Statistical tests** | |
| **Diet** | **Chow** | **HFHS** | **Chow** | **HFHS** | **Chow** | **HFHS** | **Chow** | **HFHS** | **Effect of Time** | **Effect of Diet** |
| **Stomach Weight (g)** | 2.2±0.1 | 2.4±0.1 | 4.1±0.4 | 3.8±0.5 | 5.7±1.1 | 5.5±0.5 | 5.2±0.6 | 3.9±0.6 | **** | ns |
| **Plasma Glucose (mM)** | 9.2±0.3 | 9.8±0.6 | 10.3±0.3 | 9.7±0.3 | 10.5±0.2 | 10.6±0.3 | 9.7±0.1 | 10.7±0.5 | * | ns |
| **Liver R_GIG_**  **(μmol 100g^-1^min^-1^)** | 2.7±0.3 | 2.4±0.5 | 3.7±0.3 | 4.5±0.8 | 3.8±0.7 | 2.9±0.3 | 3.1±0.3 | 3.2±0.3 | ** | ns |
| **Liver Glycogen**  **(μmol/g)** | 77.3±10.9 | 59.7±10.8 | 100.7±11.5 | 140.0±12.0 | 153.8±14.1 | 183.3±9.0 | 305.3±17.5 | 235.6±23.7 | **** | ns |
| **TC R_GIG_**  **(μmol 100g^-1^min^-1^)** | 1.3±0.2 | 0.9±0.2 | 2.2±0.3 | 2.2±0.4 | 2.9±0.6 | 2.5±0.2 | 1.8±0.2 | 1.6±0.2 | **** | ns |
| **TC Glycogen**  **(μmol/g)** | 39.5±1.1 | 42.9±1.4 | 40.5±1.4 | 38.0±2.7 | 43.1±1.8 | 40.4±2.8 | 41.6±1.8 | 41.3±2.1 | ns | ns |
| **RQ R_GIG_**  **(μmol 100g^-1^min^-1^)** | 2.6±0.4 | 1.9±0.6 | 5.2±0.9 | 4.9±1.3 | 7.4±2.7 | 4.9±0.4 | 7.0±0.8 | 3.8±0.6 | ** | * |
| **RQ Glycogen**  **(μmol/g)** | 51.4±1.4 | 52.7±3.3 | 51.8±2.2 | 58.2±6.0 | 50.4±1.6 | 53.9±2.5 | 50.7±2.0 | 60.7±10.0 | ns | ns |
| **WQ Rg’**  **(μmol 100g^-1^min^-1^)** | 2.2±0.2 | 2.5±0.2 | 3.3±0.2 | 4.9±0.9 | 4.0±0.7 | 3.8±0.3 | 3.9±0.7 | 4.3±0.6 | ** | ns |
| **EpiWAT Rg’**  **(μmol 100g^-1^min^-1^)** | 0.8±0.1 | 0.5±0.1 | 0.8±0.1 | 0.8±0.2 | 0.9±0.1 | 1.0±0.1 | 1.1±0.2 | 0.6±0.1 | p = 0.07 | ns |
| **IngWAT Rg’**  **(μmol 100g^-1^min^-1^)** | 1.6±0.3 | 1.5±0.4 | 3.2±0.5 | 2.3±0.4 | 2.0±0.4 | 2.4±0.6 | 2.6±0.5 | 2.0±0.4 | ns | ns |
| **Heart Rg’**  **(μmol 100g^-1^min^-1^)** | 44.2±7.0 | 28.5±4.3 | 80.4±7.4 | 50.1±6.8 | 69.8±6.5 | 48.9±2.7 | 70.8±9.6 | 48.8±2.9 | *** | **** |
| Values are means ± SEM. Analyzed by 2-way ANOVA for an effect of time and an effect of diet. * p < 0.05, ** p < 0.005, *** p < 0.0005, **** p < 0.0001. ns = not significant. n = 5-10. TC = tibialis cranialis. RQ = red quadriceps. WQ = white quadriceps. EpiWAT = epididymal white adipose tissue, Ing = inguinal. R_GIG_ = rate of glucose incorporation into glycogen. Rg’ = rate of glucose uptake. | | | | | | | | | | |

Table S4: **Excel spreadsheet of complete proteomics and phosphoproteomic datasets.** Expression values are log2 fold change compared to a shared standard (pooled RQ from 3, 6-hour fasted chow-fed rats). Spreadsheet 1 = phosphopeptides. Spreadsheet 2 = Phosphopeptide Pearson correlations (after inverse log transformation) with plasma insulin (mU/L) and glucose uptake (Rg’, umol min^-1^100g^-1^). Spreadsheet 3 = total proteomics.
